# Whey Protein Isolate-Chitosan PolyElectrolyte Nanoparticles as a Drug Delivery System

**DOI:** 10.3390/molecules28041724

**Published:** 2023-02-11

**Authors:** Zahra Yadollahi, Marjan Motiei, Natalia Kazantseva, Jaroslav Císař, Petr Sáha

**Affiliations:** 1 Centre of Polymer Systems, Tomas Bata University in Zlín, Třída Tomáše Bati 5678, 76001 Zlín, Czech Republic; 2 Faculty of Technology, Tomas Bata University in Zlin, Vavrečkova 275, 76001 Zlín, Czech Republic

**Keywords:** WPI, chitosan, TPP, colloidal stability

## Abstract

Whey protein isolate (WPI), employed as a carrier for a wide range of bioactive substances, suffers from a lack of colloidal stability in physiological conditions. Herein, we developed innovative stabilized PolyElectrolyte Nanoparticles (PENs) obtained by two techniques: polyelectrolyte complexation of negatively charged WPI and positively charged chitosan (CS), and ionic gelation in the presence of polyanion tripolyphosphate (TPP). Therefore, the WPI-based core was coated with a CS-based shell and then stabilized by TPP at pH 8. The nanostructures were characterized by physiochemical methods, and their encapsulation efficiency and in vitro release were evaluated. The spherical NPs with an average size of 248.57 ± 5.00 nm and surface charge of +10.80 ± 0.43 mV demonstrated high encapsulation efficiency (92.79 ± 0.69) and sustained release of a positively charged chemotherapeutic agent such as doxorubicin (DOX). Z-average size and size distribution also presented negligible increases in size and aggregates during the three weeks. The results obtained confirm the effectiveness of the simultaneous application of these methods to improve the colloidal stability of PEN.

## 1. Introduction

Nanobiotechnology is a new discipline connecting physical and biological sciences to create new tools for comprehending biological systems, diagnosing diseases, and treating patients [1,2]. Drug delivery systems in nanobiotechnology are essential for prevention and disease treatment, especially in cancer [3]. A significant variety of drug delivery vehicles, such as polyelectrolyte nanoparticles (PENs), have been investigated to improve anticancer therapeutic safety and efficacy [4]. PENs are a kind of assembly between oppositely charged drug–polymer, polymer–polymer, or polymer–drug–polymer [5]. The main driving forces are associated with electrostatic, van der Waals, hydrophobic, and hydrogen bond interactions [5]. These complexes exhibit desirable physicochemical properties of various polymers and possess the advantage of simple preparation [6,7]. Herein, natural food-grade materials such as whey protein (WP) and chitosan (CS) are utilized in constructing PENs as promising and versatile nano-delivery systems (NDS).

WP, a by-product of cheese processing, has gained great attention as an NDS due to its biocompatibility, biodegradability, low cost, and toxicity. These NDS have demonstrated a variety of functionalities, including high encapsulation efficiency, sustained release behavior, and rapid absorbance across biological membranes [8,9]. Whey protein isolate (WPI) is filtered WP that contains more proteins (i.e., α-lactalbumin and β-lactoglobulin). Amphiphilic WPI with a pKa of 4.9 and functional groups on the primary structure has been employed to improve the bioavailability and stability of hydrophobic nutrients and deliver a wide range of bioactive substances at particular target sites [10,11]. However, it has drawbacks, such as thermal, pH, and ionic strength sensitivity, which can be improved by integrating it into other polymeric structures [11,12]. Therefore, this study aims to fabricate PENs by integrating WPI with CS as a drug delivery system. 

CS is a cationic biopolymer of N-acetylglucosamine and D-glucosamine units, and is widely employed in theoretical medicine and food technology due to its mucoadhesive, biocompatibility, biodegradability, non-toxicity, and antibacterial properties [13,14]. CS shows a positive charge below its pKa of 6.5 through the protonation of amino residues, which is the basis of the immobilization of negatively charged molecules such as polymers, proteins, medicines, and other cargoes [15]. CS forms polyplex aggregation [16] or coacervation [17] in the presence of polyanions such as tripolyphosphate (TTP). TTP is a nontoxic, weak polyprotic acid and multivalent anionic cross-linker with a pKa of 0.89. It can improve the quality of electrostatic interactions, which leads to the formation of PENs and a sustained release rate [18,19]. These polyplexes can be employed for various purposes, including micro- and nano-encapsulation of pharmaceuticals and chemicals [7,20], as well as magnetic nanoparticles, which are successfully used as drug delivery agents by application of an external magnetic field [21]. 

There are different studies on WPI-CS polyplexes, such as WPI-CS film in food packaging [22,23] and NPs as delivery systems [24,25]. They utilized different techniques, including nanoprecipitation [24,26,27], oil-in-water (O/W) emulsion [25], and co-assembly [11,28,29] to synthesize WPI-CS constructions. Nonetheless, this study aims to fabricate an innovative WPI-CS NP through two techniques, including polyelectrolyte complexation and ionic gelation, to self-assemble the anionic WPI with a cationic CS in the presence of TPP. Therefore, WPI was utilized at a pH value higher than its pKa (4.9), and CS at a pH value lower than its pKa (6.5). Finally, the NPs are utilized for loading doxorubicin (DOX) as a model drug with a pKa value of 9.93 [30]. Alves et al. confirmed that 80% of DOX is in the cationic form at pH 7.4, which predicts driving forces such as electrostatic interaction with the donors [30]. These driving forces stabilize the polyelectrolyte complexes [6,31], and these stabilized particles protect DOX from the harsh environment of cancerous tissue (i.e., pH and thermal conditions) [32]. 

## 2. Results

### 2.1. Effect of pH and Concentration on Hydrodynamic Size

**Figure 1:**
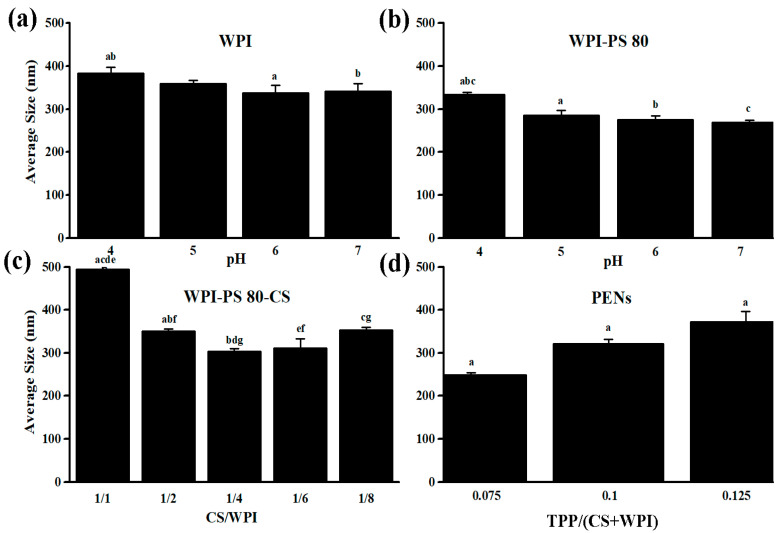
The relationship between z-average size to (**a**) pH of WPI, (**b**) pH of WPI in the presence of PS80, (**c**) CS/WPI ratio, and (**d**) TPP concentration. *n* = 3, mean ± standard deviation, the same letters indicate significant differences between the means of size (*p* value < 0.05), and the values marked with the different letters are not statistically different.

Due to the crucial role of NP size on therapeutic efficacy (i.e., long circulation, biodistribution, and clearance), the size distribution of NPs was first explored at different pHs and polymer concentrations. pH is one of the primary characteristics that impact the electrostatic interactions among the ionized functional groups and, subsequently, the size distribution (i.e., particle size and polydispersity index (PDI)) and ζ-potential [33]. The size distribution, among other parameters, determines the suitability of the formulations for a particular route of drug administration [34]. The particle size of about 80 to 300 nm is beneficial for cancer cell internalization through endocytosis pathways because the pore size of the tumor vessel varies from 200 nm to 1.2 µm depending on the tumor type [35]. The PDI provides information about the heterogeneity of the size distribution of particles. PDI values smaller than 0.5 mainly show highly monodisperse standards. PDI values greater than 0.7 indicate a broad particle size distribution [34]. Therefore, the impact of pH on the size distribution of WPI was first assessed. According to Figure 1a, there is no significant difference among the size distribution of WPI at pH values of 5 (359.83 ± 7.67 nm and 0.59 ± 0.09), 6 (338.07 ± 17.35 nm and 0.59 ± 0.10) and 7 (341.30 ± 17.78 nm and 0.57 ± 0.09) as opposed to pH 4 (383.23 ± 14.08 nm and 0.58 ± 0.15). WPI above or below its isoelectric point (pI) of 4.9 shows shifting in its ionized and unionized forms and increasing repulsive electrostatic forces, which plays an essential role in unfolding and reducing self-aggregation [20]. After that, the average size of the particles was evaluated in the presence of Polysorbate 80 (PS 80) at different pH values, which displays significant differences at pH 4 to pH values 5, 6, and 7. The smallest size and relatively narrow distribution of WPI (268.28 ± 51.68 nm and 0.76 ± 0.20) were observed at the pH of 7 (Figure 1b). PS 80 significantly reduced WPI size at pH 7 and a concentration of 0.5%, confirming that co-solvents’ actions are extremely pH- and concentration-dependent [20]. As shown in Figure 1c, in the presence of CS with pH values of 5.0, significantly small particles were obtained at the CS: WPI *w*/*w* ratio of 1:4 (302.73 ± 6.82, 0.61 ± 0.11) [11]. However, these findings confirmed that, aside from pH, the biopolymer ratio significantly impacted the average size of the PENs [11]. Finally, the PENs were synthesized in the presence of TPP at the TPP:(CS+WPI) *w*/*w* ratio of 0.075, 0.1, and 0.125 at pH 8, which affected the hydrodynamic size considerably (Figure 1d). The smallest size of the particles was about 248.57 ± 5.00, with a PDI of 0.41 ± 0.02. At pH 8, TPP is dissociated into OH^−^ and TPP ions (HP_3_O_10_^4−^ and P_3_O_10_^5−^). OH^−^ produces a coacervation barrier, which leads to lower diffusion of P_3_O_10_^5−^ into CS. Despite lowering the net positive charge of CS by OH^−^, it has sufficient binding sites for diffused P_3_O_10_^5−^. Finally, P_3_O_10_^5−^ ions interact electrostatically with positive amino groups of CS and overcome repulsive forces among positive functional groups of CS chains [36,37].

The ζ-potential is another important factor that influences the physicochemical properties of PENs. The ζ-potential, or electrokinetic potential, is an index for particle surface charge and stability [38,39]. The repulsive interactions become stronger as the ζ-potential increases, resulting in more stable particles with a more uniform size distribution. A physically stable PEN will have a minimum ζ-potential of ±30 mV [40], which is critical in aggregate prevention. The ζ-potential of WPI was around −19.57 ± 0.70, −22.50 ± 0.92, −23.57 ± 1.17, and −26.83 ± 1.30 at different pH values ranging from 4 to 7. After that, the ζ-potential slightly increased in the presence of PS 80 to −23.83 ± 0.058, confirming the stabilizer’s effect on the surface charge of the PENs. Wilson et al. also demonstrated that poly(n-butyl cyanoacrylate) NPs coated with 1% PS 80 had a higher average ζ-potential than NPs without coating [40]. PS 80 is a surfactant that can alter the particle ζ-potential by decreasing electrostatic repulsion, stabilizing suspensions, and decreasing particle aggregation. However, increasing the PS 80 concentration generally leads to a decrease in ζ-potential [41]; PS 80 effect depends on various factors, such as the PS 80 concentration, pH, ionic strength, and the nature of the particles [42]. The ζ-potential value increased to 22.97 ± 1.08 after adding CS to the core structure. As a result of the electrostatic interactions between the free primary amino groups of CS and the anionic groups of the WPI, the *ζ*-potential increased [11]. Finally, due to the presence of TPP ions, which interacted with the CS backbone and decreased the number of free amino groups, the smallest PENs with the most uniform distribution showed a *ζ*-potential of 10.80 0.43 mV. This *ζ*-potential value is adequate for forming stable, suspended NPs [40]. Moreover, the final optimized formulation contained a CS:WPI *w*/*w* ratio of 1:4 and a TPP:(CS+WPI) *w*/*w* ratio of 0.075 at pH values of 7.0 (WPI), 5.0 (CS), and 8.0 (TPP), which were utilized for further analysis.

### 2.2. Morphology of PENs

**Figure 2:**
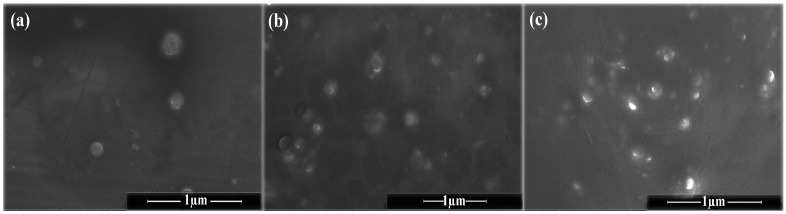
SEM images of (**a**) WPI, (**b**) WPI-CS, and (**c**) PENs in a dried state.

SEM micrographs of PENs are shown in Figure 2. These images showed spherical particles with an average size of 329.10 ± 65.24 nm (WPI), 399.91 ± 55.56 nm (WPI/CS), and 256.26 ± 34.52 nm (PENs), which is consistent with DLS data. WPI particles identified by SEM analysis were smooth, spherical, and more apparent in shape (Figure 2a). In contrast, the addition of CS changed the particle morphology to particles with vacuoles of various sizes scattered across them (Figure 2b), as confirmed by Huang et al. [43]. Finally, in the presence of TPP, the PENs showed more compact structures with rough edges (Figure 2c), which might be attributed to the complete cross-linking of CS by the polyanionic TPP [6].

### 2.3. Colloidal Stability Analysis

Colloidal stability is one of the main factors determining the appropriateness of a particular route for drug delivery. Therefore, controlling this variable is crucial for the successful clinical use of NPs [34]. In this construction, due to the low conformational stability of proteins, their complexation by polysaccharides through electrostatic interactions can lead to new rheological behaviors with high stability [44]. Herein, anionic WPI was coated by a cationic low-MW CS in the presence of TPP, and the stability was evaluated by DLS upon storage at 4 °C for one month. The average particle size and the PDI are the most common indicators of particle quality in size distribution. Different researchers utilized average particle size and PDI to evaluate the colloidal stabilities of NPs [45,46,47]. According to Table 1, the sample presented negligible increases in size (261.87 ± 9.16) and aggregates confirmed by a PDI value of 0.49 ± 0.04 for three weeks. The particle size of around 80 to 300 nm is beneficial for endocytosis in cancerous cells because the pore size of the tumor vessel varies from 200 nm to 1.2 µm depending on the tumor type [35]. The PDI provides information about the degree of heterogeneity of particles. PDI values smaller than 0.5 mainly show highly monodisperse standards [34]. Therefore, the low PDI during the 22 days indicates a homogeneous dispersion. It can be deduced that the stabilization of the nanosystem occurred through electrostatic interactions or hydrogen bonds among the biopolymers by adjusting pH and ratio. Nonetheless, PENs started to swell and aggregate in the fourth week, as confirmed by a significant increase in z-average size (345.03 ± 44.64) and PDI value of 0.80 ± 0.21 (Table 1). PDI values greater than 0.7 indicate broad particle size distribution and aggregation [34]. It can be concluded that a water inflow to the PENs happened, which increased their size significantly [6].

### 2.4. Infrared Spectrophotometry Analysis of PENs

**Figure 3:**
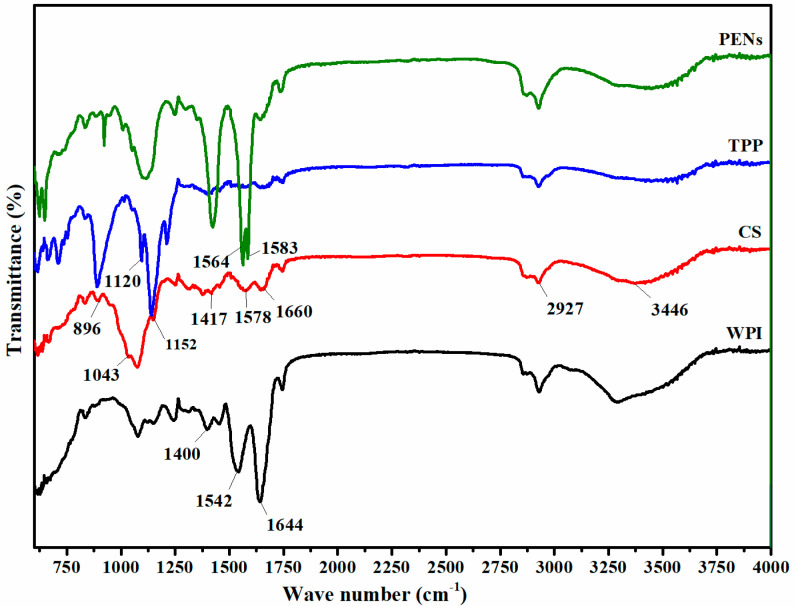
FTIR spectra of WPI, CS, TPP, and PENs.

To assess the occurrence of intermolecular interactions in WPI-CS complexation, FTIR-ATR analysis was conducted at a wavenumber of 400–4000 cm^−1^ (Figure 3). The representative peaks of the WPI backbone are as follows: the stretching of C=O at 1644 cm^−1^ (amide I), the bending of N-H (amide II) at 1542 cm^−1^, and the N-H bending and C-N stretching vibrations (amide III) at 1400 cm^−1^ [8]. The IR spectrum of CS showed the peaks of amid I at 1660, amid II (NH_3_^+^ groups) at 1578, an amino functional group at 3446 cm^−1^, the extension of the vibration of the C-H bond at 2927 cm^−1^, the vibration bands of -OH and -CH groups at 1417 cm^−1^, the symmetrical stretching of C-O-C at 1152 cm^−1^, vibrational stretching of C-O at 1043 cm^−1^, and the pyranose ring at 896 cm^−1^ [31]. Finally, the spectra changes in NH_3_^+^ groups of CS (1578 cm^−1^), the C=O stretching (1644 cm^−1^), the N-H bending (1542 cm^−1^) of WPI, P=O band of TPP (1120 cm^−1^), and the appearance of two strong stretching bands at 1583 and 1564 cm^−1^ in the COO^−^ antisymmetric region of PENs confirmed electrostatic interaction between the amine groups of CS (NH_3_^+^) and the carboxyl groups of WPI (COO^−^). In addition, the spectra of the PENs showed a broader band at around 3000–3600 cm^−1^ compared to the spectra of WPI and CS, which demonstrated hydrogen bonding [8]. The C=O stretching vibrations at 1600–1700 cm^−1^ also declared the intensity of hydrogen bonds and interactions across amide units on the protein structure [32]. These findings suggest the presence of hydrogen bonding in addition to electrostatic interactions in the formation of PENs.

### 2.5. Thermogravimetric Analysis 

The thermal stability of the pure materials and PENs was evaluated by determining their total percentage weight loss at 25 °C to 600 °C (Table 2).

**Figure 4:**
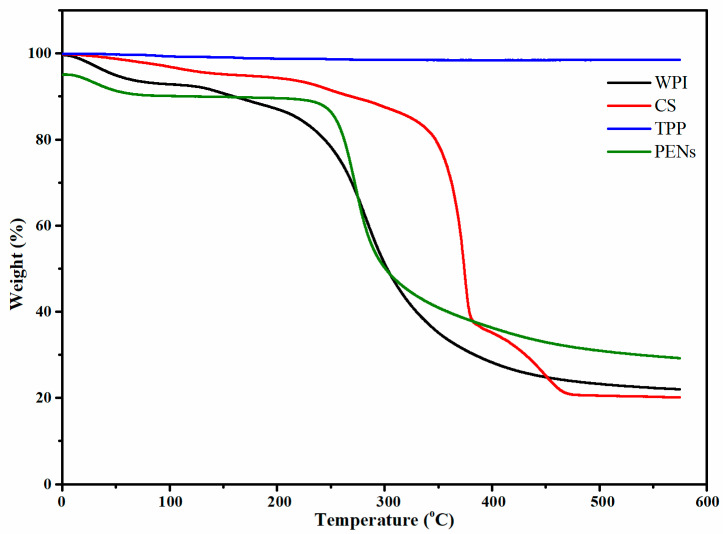
Thermogravimetric analysis of WPI, CS, TPP, and PENs.

As shown in Figure 4, the regular diminishment of the weights below ∼100 °C was mainly due to free and bonded CO_2_, H_2_O, and other gases. CS lost about 60% of its mass from 293.34 °C to 594.88 °C due to de-polymerization and the loss of amino and CH_2_OH moieties [33]. Other WPI thermal mass loss events occurred at Tmaxes of 164.36 °C and 303.20 °C. Tmax of 164.36 °C can be attributed to the de-polymerization of WPI by breaking the peptide linkages, resulting in a weight loss of 4.86%. The last weight loss of 65.81% happened between 210.78 °C and 591.65 °C due to protein decomposition [34]. The TGA curves of PENs showed four primary thermal degradation and weight loss zones. Firstly, weight loss behavior of approximately 5.00% at temperatures ranging from 25 to 122.45 °C was related to the diminishment of freezing-bound water. A further increase to 265.72 °C resulted in weight losses of 5.52% attributed to covalent peptide bonds, and the third stage at 265.72–394.84 °C with the highest degradation rate of 53.73% is correlated to the degradation of CS and WPI. The final stage, with a weight loss of 15.15%, was 394.84 to 471.35 °C associated with the complete degradation of organic compounds. Moreover, the PENs had a higher Tmax than their components, which can be attributed to the higher amount of water trapped in the nanostructure.

### 2.6. Drug Loading Assay of PENs 

In this stage, the impact of polyelectrolyte structure on the loading capacity and release behavior of Dox-loaded PENs was examined. The results showed an EE (%) and LC (%) of around 92.79 ± 0.69 and 4.12 ± 0.03, respectively. Different factors affect EE and LC, including the chemical structure of the drug (i.e., functional groups), the core (i.e., length and functional groups), the shell (MW and functional groups), and the cross-linker (i.e., pH and concentration) [33]. Zhang et al. also confirmed that the environment significantly influences drug loading performance [45]. Therefore, the presence of oppositely charged groups in the chemical structures of DOX, WPI, CS, and TPP leads to strong electrostatic interactions, and the nanostructure displayed high amounts of EE. Nonetheless, the low amount of LC (%) can be explained by the strong dependency of LC on the weight ratio of NPs, in accordance with Equation (1), Section 3.4.

### 2.7. In Vitro Release Study of PENs

**Figure 5:**
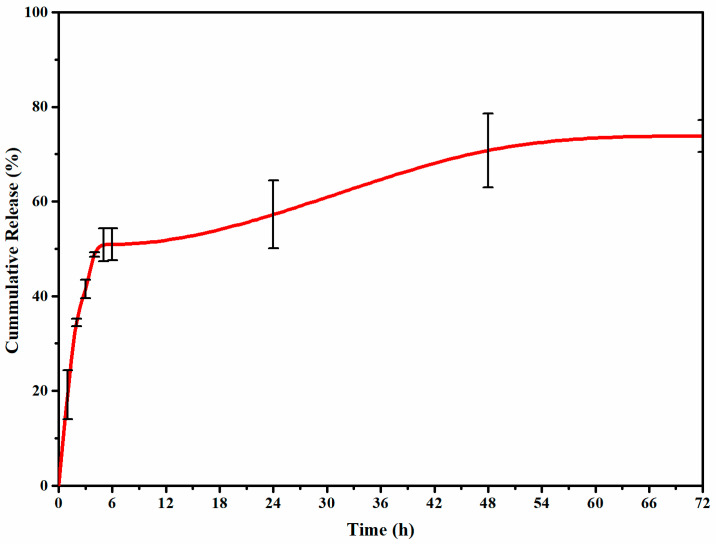
In vitro release profiles of DOX-loaded PENs at physiologic pH of release media (7.4).

The in vitro release profile in Figure 5 demonstrated a two-step biphasic process with an initial burst release for 4 h and a subsequent steady release for 72 h. Noncovalent interactions, including hydrophobic, electrostatic, and hydrogen bonding, are critical in improving drug release. The cross-linking pH and concentration, carrier structure, and pH and temperature of the release medium can influence these interactions. During the initial burst release, adsorbed or trapped DOX molecules on the polymer surface coatings are released into the media. The subsequent slower release can be mainly attributed to DOX encapsulated in the WPI core structure, which can be described by the physical barriers (i.e., WPI core and CS shell) and strong self-assembly of functional groups between DOX-WPI, CS-WPI, and TPP-CS. These interactions can be reversible electrostatic interactions, van der Waals interactions, hydrophobic interactions, and hydrogen bonds [46]. According to Mattu et al., the interaction of fully protonated amino-binding sites of the shell (CS) with TPP dissociated into OH¯, HP_3_O_10_^4−^ and P_3_O_10_^5−^ resulted in a more compact structure and a lower release rate [47]. As a result, in addition to the electrostatic interaction of WPI-DOX, the addition of CS and TPP influences the release rate by improving inter- and intramolecular forces.

## 3. Materials and Methods

### 3.1. Materials 

WPI (protein ˃ 71.0% and ash ˂ 6.0%) was supplied by MEGGLE (Wasserburg am Inn, Germany). Low molecular weight CS (MW of 50–190 kDa, degree of deacetylation (DD) ≥ 75%), DOX, PS 80, TPP, dimethyl sulfoxide (DMSO), and dialysis tubing with a cutoff of 12 kDa MWCO were purchased from Sigma-Aldrich (St. Louis, MO, USA). VWR Chemicals (Stříbrná Skalice, Czech Republic) supplied the other chemicals, which included acetic acid, sodium chloride, di sodium hydrogen phosphate, potassium chloride, and potassium dihydrogen phosphate.

### 3.2. Preparation of PENs

CS was dissolved in 1% acetic acid to form a homogeneous solution. After adjusting the pH to 5, PS 80 (0.5%) was added to the CS solution (1 mg/mL, pH 5) as a nonionic steric stabilizer at 500 rpm for 1 h. WPI solution was also prepared in a 1 mg/mL concentration in dH_2_O at different pH values of 4–7 while stirring for 1 h. Following that, different CS: WPI solution ratios (i.e., 1/1, 1/2, 1/4, 1/6, and 1/8) were prepared by adding WPI solution to CS solution dropwise and stirring for 1 h.

At this stage, WPI and CS were complex through electrostatic interactions. Then, TPP (1 mg/mL, pH 8) was added to the solution at different *w*/*w* ratios of 0.05, 0.075, and 0.1 while stirring at 1000 rpm for 30 min. The CS-TPP solution was subjected to ionic gelation through cross-linking CS chains with TPP. Finally, the optimized formulation was characterized by dynamic light scattering (DLS), scanning electron microscopy (SEM), Fourier transform infrared spectroscopy (FT-IR), and thermogravimetric analysis (TGA).

### 3.3. Characterization of PENs

The average diameter, polydispersity index (PDI), and ζ-potential of PENs were determined by DLS (Nano ZS, Malvern, UK) at 25 °C by disposable polystyrene cuvettes and Malvern ζ-potential disposable folding capillary cuvettes, respectively. The surface morphology of the PENs was examined using a Nova450 NanoSEM, FEI (Brno, Czech Republic) at an elevated voltage of 10 kV. The dried samples on a sheet of aluminum foil were attached to the SEM specimen stub using a double-sided carbon adhesive disc (Taab, Aldermaston, UK) and then coated by gold/palladium sputtering (SC7620 Mini Sputter Coater, Quorum Technologies, Laughton, UK, 10 mA for 45 s). The Nicolet iS5 FTIR spectrometer analyzed the specific functional groups of the raw materials and nanostructure with the iD5 ATR accessory Ge crystal at a resolution of 4 cm^−1^ and 64 scans in the wavelength region of 4000–600 cm^−1^. Finally, the thermogravimetric analysis was carried out using a TA Instruments Q500 Thermogravimetric Analyzer (Wilmington, USA) at a heating rate of 10 °C min^−1^ in a nitrogen atmosphere between 25 and 600 °C. The Universal Assessment 2000 system was then utilized to calculate the weight loss percentage of the components.

### 3.4. Drug Loading Assay of PENs

In order to fabricate DOX-loaded PENs, DOX at a concentration of 0.04 mg/mL was first added to the WPI solution under vigorous stirring for 30 min, and then CS and TPP were added, as mentioned before. Drug loading of DOX-loaded PENs was assessed by a dialysis method and in the presence of phosphate-buffered solution (PBS, 10 mM, pH = 7.4). A dialysis tube containing DOX-loaded PENs solution (11 mL) was placed into 100 mL of PBS to assess encapsulation efficiency (EE) and loading capacity (LC). Then, the entire system was kept in an orbital incubator (Stuart SI500, UK) at 37 ± 0.5 °C, 40 rpm for 1 h. EE (%) and LC (%) were calculated according to Equations (1) and (2), respectively [33], where Total DOX was the quantity of primary DOX and Free DOX was measured by UV-vis spectrophotometry (CARY 300 Conc, USA) at 480 nm versus a calibration curve (R^2^ = 0.998, *n* = 3).

(1) $$\mathrm{LC}\left(\%\right)=\frac{\mathrm{Total}\;\mathrm{DOX}-\mathrm{Free}\;\mathrm{DOX}\;}{\mathrm{Nanoparticle}\;\mathrm{Weights}}\times 100$$

(2) $$\mathrm{EE}\left(\%\right)=\frac{\mathrm{Total}-\mathrm{Free}\;}{\mathrm{Total}\;\mathrm{DOX}}\times 100$$

### 3.5. In Vitro Release Study of PENs

In order to assess the in vitro release rate of DOX-loaded PENs, the dialysis tube containing the DOX-loaded PENs’ solution was placed into a 50 mL PBS container. The entire system was placed in an orbital incubator for 72 h at 37 ± 0.5 °C and 40 rpm. A total of 3 mL of the medium was taken at regular intervals, and the same volume of the fresh medium was introduced to the system. UV-vis spectrophotometry was utilized to measure the concentration of DOX in the medium compared to a calibration curve at 480 nm for in vitro drug release testing of formulation.

### 3.6. Statistical Analysis

Each experiment was performed in triplicate, and the results were presented as the mean ± standard deviation. The statistical analysis was performed through Microsoft Excel. A one-way ANOVA was used to compare the means among the groups, and a Student’s *t*-test was used to compare mean data between each group. The differences were considered significant when *p* < 0.05 was used.

## 4. Conclusions

Novel PENs were synthesized through polyelectrolyte complexation and ionic gelation techniques utilizing a WPI-based core, a CS-based shell, and an ionic cross-linked polyanion, TPP. The vacuolated spherical core/shell nanostructures showed high colloidal stability at the predetermined time intervals for three weeks. The particle complexation and thermal stability were confirmed by ATR-FTIR and TGA, respectively. The PENs also demonstrated high encapsulation efficiency and sustained release rate of DOX at physiological pH, governed significantly by a high amount of hydrogen bonds and electrostatic interactions between WPI and DOX. These results offer a promising drug delivery system with desirable stability and prolonged systemic circulation that should be developed.

## Figures and Tables

**Table 1:** Comparison of PENs’ z-average size and PDI by DLS upon storage at 4 °C for one month.

Day	Z-Average Size	PDI
1	248.57 ± 5.00 ^a^	0.41 ± 0.02 ^e^
8	253.10 ± 6.21 ^b^	0.44 ± 0.06 ^f^
15	261.20 ± 8.35 ^c^	0.46 ± 0.05 ^g^
22	261.87 ± 9.16 ^d^	0.49 ± 0.04 ^h^
29	345.03 ± 44.64 ^abcd^	0.80 ± 0.21 ^efgh^

*n* = 3, mean ± standard deviation. The same letters in the same row indicate significant differences between the means of size and PDI of the particles (*p* value < 0.05), and the values marked with the different letters are not statistically different.

**Table 2:** Total percentage weight loss in pure components and PENs.

Compounds	Total Loss of Mass (%)
CS	65.87
TPP	1.456
WPI	77.48
PENs	79.41

## Data Availability

Data available on request from the authors.

## Sample Availability

Samples of the compounds are available from the authors.
